# Socio-demographic and clinical profile of immuno-histochemically confirmed breast cancer in a resource limited country

**DOI:** 10.11604/pamj.2014.17.182.2257

**Published:** 2014-03-10

**Authors:** Ganiyu Adebisi Rahman, Samuel Adegboyega Olatoke, Suleiman Olayide Agodirin, Kayode Adebanji Adeniji

**Affiliations:** 1 General Surgery Division, Department of Surgery, University of Ilorin Teaching Hospital Ilorin, Ilorin, Nigeria; 2 Department of Pathology, University of Ilorin Teaching Hospital Ilorin, Ilorin, Nigeria

**Keywords:** Breast cancer, immunohistochemistry, subtypes, resource limited country, Nigeria

## Abstract

**Introduction:**

Breast cancer is the most common cancer in females. It is the most common cause of cancer-related death among women with fatality rates highest in low-income countries. The aim of this study is to determine the socio-demographic and clinical profile of patients with immunohistochemically confirmed breast cancer in a Nigerian tertiary health institution.

**Methods:**

Patients with immunohistochemically confirmed breast cancer were reviewed. The information retrieved was entered into a proforma designed for the purpose of the study. Data was analysed using SPSS version 18.0.

**Results:**

The peak incidence of age at presentation was in the 5th decade. More than 50% of the patients were premenopausal and perimenopausal at presentation. Only 11% of the patients presented with breast lumps less than 2 cm in size. Women in the age group 50-59 years are more likely to present with larger breast lumps than women in other groups. More than 50% had clinically palpable lymph node at presentation. Mastectomy (simple mastectomy and modified radical mastectomy) and adjuvant chemotherapy were the main form of treatment. Most of the cases were estrogen receptor negative with majority of them having basal-like subtype.

**Conclusion:**

Most of the patients in this study were not only young but presented with locally advanced disease. Population screening, adequate health education, improved accessibility and availability of heath care will go a long way to improve the outcome of these patients.

## Introduction

Breast cancer is the commonest form of cancer world wide [1]. There is a change in pattern of disease in developing countries with the emergence of non-communicable disease along side the resurgence of some previously controlled infectious diseases. As at 1985, of an estimated 9 million new cases of cancer each year, 4 million were in developed and 5 million in developing countries; by 2015, these figures are likely to reach 15, 5, and 10 million respectively. From these figures an increase of 25% will take place in developed countries with 100% in developing countries [2].

## Methods

The clinical records of patients who presented to the University of Ilorin Teaching hospital, Ilorin from 2003-2008 were reviewed. University of Ilorin Teaching Hospital is a 500 bed tertiary health institution in Nigeria. Information was retrieved from the case notes, operation records and histopathology records. The information extracted were socio-demographic (age, sex, menstrual status, parity, age at menarche and menopause), family history, associated medical condition, symptoms and signs at presentation, imaging modalities, tissue diagnosis with immunohistochemically determined subtypes, treatment and outcome of treatment. Breast cancer subtypes were defined as luminal A (ER+ and/or PR+, HER2-), luminal B (ER+ and/or PR+, HER2 + ), basal-like (ER-, PR-, HER2-, CK5/6+, and/or EGFR + ), HER2 + /ER- (HER2+, ER-, PR-), and unclassified (negative for all five markers). The information retrieved was entered into a proforma designed for the purpose of the study. Data was analysed using SPSS version 18.0.

## Results

**Figure 1:**
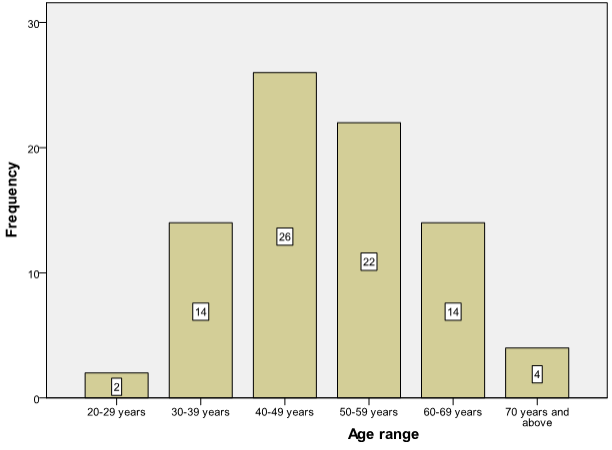
Age distribution of patients with breast cancer (n= 82)

There were more than 200 patients with breast cancer seen during the study period, but only those with detailed clinical profile and immunochemical characteristics of breast tissue were included in this study. There were 82 patients reviewed. The youngest patient in this study was 29 years old and the oldest 75 years with a mean of 48.98 (SD 10.97). The peak incidence of age at presentation was in the 5th decade of life (Figure 1). Only three (3.7%) patients were male, seventy-nine (96.3%) were females. Forty-three (52.5%) were pre-menopausal or peri-menopausal at presentation. Seventy-eight (95.1%) had no family history of breast cancer with only four (4.9%) having a family history. Two (2.5%) patients were nulliparous, five have had one child at presentation and 91% of the patients were multiparous at presentation.

Though 47.5% of the patients were postmenopausal, 64.6% had no associated medical condition. High blood pressure was the commonest associated medical condition occurring in 20.7% of the patients. All patients in this study presented with a lump. Only 8.5% and 4.9% presented with nipple discharge and breast pain respectively. In this study 4.9% had previous history of non-malignant breast disease. The primary tumour involved the right in 47 (57.3%) and the left in 35 (42.7%) patients with 69.5% in the upper outer quadrant. Most of the patients 50 (61.0%) had size of lump 2.1-5.0 cm at presentation with only nine (11.0%) patients presented with lumps less than 2 cm in size. Women in the age group 50-59 years are more likely to present with larger breast lumps (> 5 cm) than women in the other groups (p ≤ 0.0001). Forty-one (50%) had clinically palpable ipsilateral axillary lymph node at presentation while 3 (3.7%) had palpable supraclavicular nodes. Only 24 (29.3%) of the patients had diagnosis confirmed within a week of presentation in the hospital. Treatment was commenced in 80 (97.6%) patient within 4 weeks of diagnosis. Sixty-five (79.3%) had breast cancer confirmed with fine needle aspiration (FNAC), others required either incisional or excisional biopsy.

**Figure 2:**
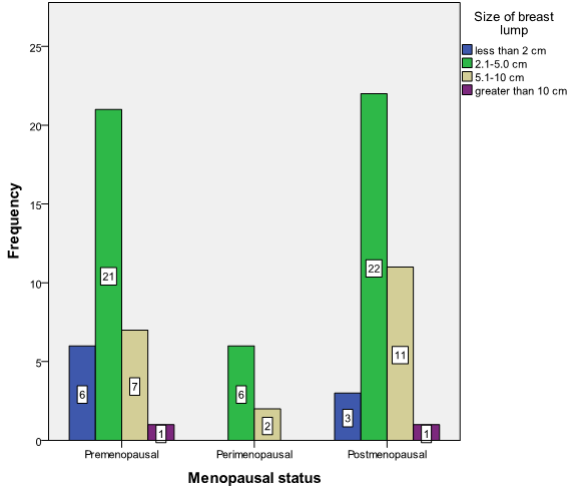
Menopausal status versus size of breast lump at presentation (n= 82)

**Figure 3:**
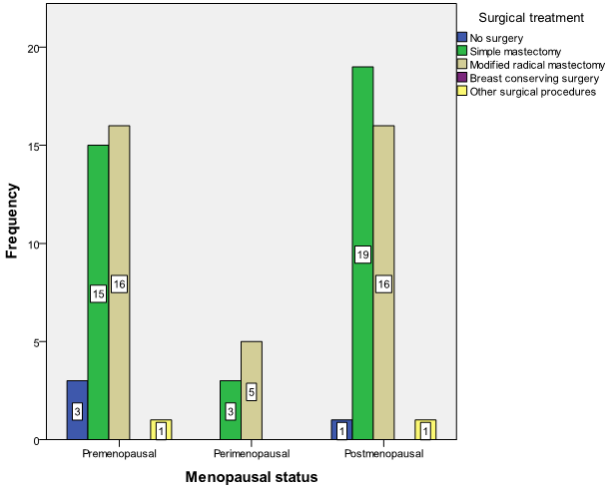
Relation between menopausal status and surgical treatment of breast cancer (n = 82)

**Figure 4:**
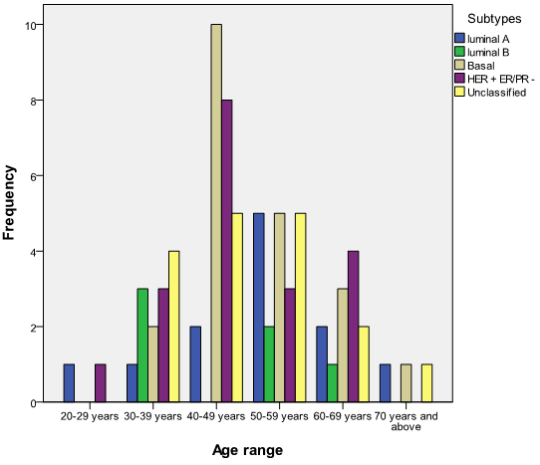
Relationship between age and subtype of breast cancer (n = 82)

**Figure 5:**
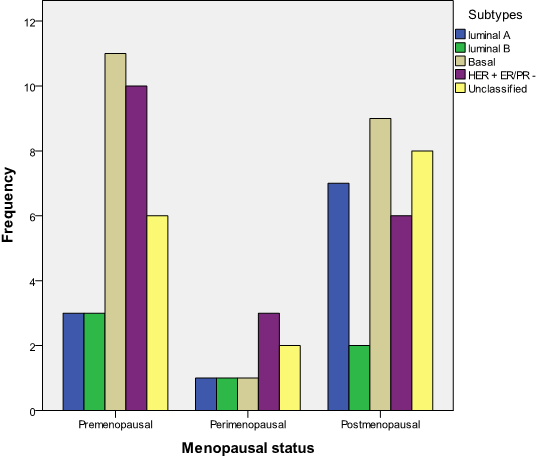
Relationship between menopausal status and subtypes of breast cancer (n = 82)

**Figure 6:**
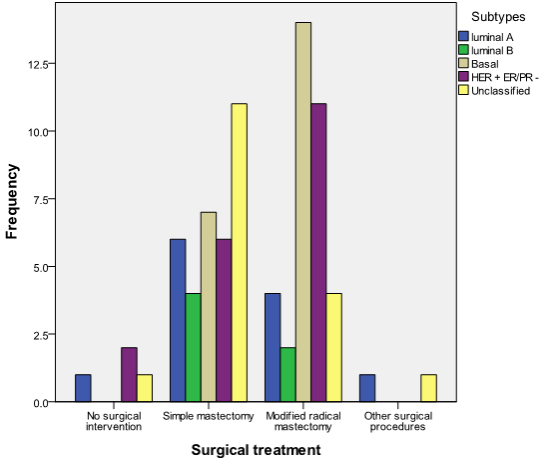
Relationship between surgical treatment and subtype of breast cancer (n = 82)

The commonest forms of surgical treatment were simple (Total) mastectomy 39 (47.6%) and modified radical mastectomy (MRM) 45.1%. Seventy-six (92.7%) patients had adjuvant chemotherapy with the use of Cyclophosphamide, Methotrexate and 5-Fluorouracil (CMF) in 53.7% of the patients and Antracycline based and Taxane based in 42.7% and 2.4% respectively. A patient refused chemotherapy as adjuvant. Figure 2 shows menopausal status versus size of breast lump at presentation while Figure 3 shows the relationship between menopausal status of patients and the type of surgical treatment given. Basal subtype is the dominant subtype in this study with the highest figures in the 5th and 6th decades of life (Figure 4). Figure 5 shows the relationship between menopausal status and the subtypes of breast cancer. Majority of the patients who had modified radical mastectomy had basal subtype while patients who had simple mastectomy had more of unclassified subtype of breast cancer (Figure 6).

## Discussion

Globally breast cancer is the commonest cancer in the females. Worldwide it is estimated that more than one million women are diagnosed with breast cancer every year, and more than 400,000 will die from the disease [3]. The incidence of breast cancer in Nigeria in 1976 was 15.3 per 100,000 but rose to 33.6 per 100,000 by 1992. Despite this doubling in incidence, many clinicians believe that there is under-reporting owing to low awareness, poor access to medical services, poverty, socio-cultural factors and absence of a screening programme. Population based epidemiological study in 1999 showed that the prevalence of breast cancer in Nigeria was 116 cases per 100,000 women per year [4].

In this study majority of the patients at presentation were young and premenopausal or perimenopausal. This is similar to studies from other African centres were the mean age is 48 years and approximately two-thirds are premenopausal [5, 6]. This is contrary to findings in Europe were majority of the women are postmenopausal [7–9]. In the area of this study there was no population screening programme at the time of the study. If a screening programme is to be introduced this age bracket 40-49 with high incidence should be taken into consideration. Though nulliparity and low parity is said to be associated with increased risk of breast cancer, in this study majority of the patients are multiparous. With basal subtype common in this study, it confirms other studies that have shown that the higher the parity is associated with triple negative breast cancer [10, 11]. It is difficult to explain this because triple negative breast cancer is not responsive to these sex hormones associated with parity.

Only 11% of the patients presented with lumps less than 2 cm in size with majority of them having clinically palpable lymph nodes. This is in keeping with other studies from developing countries where many of the patients present with locally advanced disease. In Tanzania, East Africa, more than 70% of the patients presented at stage III or IV while in Nigeria and Libya more than half present with stage III or IV [6, 12–14]. This is as a result of inadequate health education, socio-cultural belief, poverty, ignorance, lack of access to health care and use of unorthodox health care. There is also absence of population screening program. Early detection of breast cancer is not only cost-effective but also improves outcome. Unless a programmex is in place for screening and early detection, the prognosis will continue to be poor in these resource poor countries. Our patients benefited from multidisciplinary approach required especially with many of them presenting with locally advanced disease.

None of the patients in this study had breast conserving therapy (BCT) because of late presentation and also lack of radiotherapy facilities. This is because in breast conserving therapy, breast conserving surgery is followed by radiotherapy [15]. There are only 4 radiotherapy centres in Nigeria, with a population of 160 million [13]. Most of the patients had simple (Total) mastectomy, modified radical mastectomy and adjuvant chemotherapy. Women in the age group 50-59 years are more likely to present with larger breast lumps (> 5 cm) than women in the other groups (p = < 0.0001). They therefore had neoadjuvant chemotherapy before surgery.

Majority of the patients who had modified radical mastectomy had basal-like subtype of breast cancer. Mastectomy was usually followed by chemotherapy with or without radiotherapy. This is because the patients do not benefit from hormonal manipulation. The commonest subtype in this study is basal-like. This is similar to other studies from resource poor countries.

Systemic therapies are known to improve breast cancer survival. In this study many of the patients required systemic therapy. Unfortunately like other developing countries chemotherapy requires some allocation of resources and infrastructure which may not be readily available. Most of our patients had CMF regimen because it was the cheapest available at the time of this study.

## Conclusion

Breast cancer is the commonest cancer in Nigerian women. As a result of poverty and ignorance among other factors, our patients present late usually with locally advanced disease. This is further compounded by many of the patients having basal-like subtype on immunohistochemistry. There is an urgent need to have in place a population screening program for early detection, objective poverty alleviation program and improved accessible and affordable health care delivery service.
